# CVIR Special Issue on Radiation Protection

**DOI:** 10.1007/s00270-021-02816-2

**Published:** 2021-03-17

**Authors:** Werner R. Jaschke

**Affiliations:** Department of Radiology, Medical University of Innsbruck, Anichstrasse 35, 6020 Innsbruck, Austria

Radiation-induced tissue reactions and malignant diseases were for a long time considered to be an issue of the past. However, with the introduction of CT and fluoroscopically guided interventions (FGI), nearly forgotten detriments of ionizing radiation have returned. In the 1990s, several authors reported skin injuries in patients undergoing fluoroscopy-guided interventions, loss of hair following CT perfusion scans and neurointerventions [1]. Just recently, Li et al. [2] reported an alarmingly high effective dose in a significant number of patients who had one or more fluoroscopically guided interventional procedures. This calls for a change in the practice of interventional radiologists and endovascular therapists, otherwise we would be scrutinised by competent authorities and the public more and more. Since stochastic risks take several years to manifest, the connection between it and an intervention is no longer that apparent.

Smoking was for a long time an accepted habit, but it no longer is. Since a lot of data on negative health effects in smokers and people exposed to cigarette smoke was published, public opinion turned against it. This was only possible because awareness campaigns were successful and they gradually changed people’s attitude towards smoking. Nowadays, smoking is banned in public places in most countries. Like raising attention about smoking, in interventional radiology we must continuously raise the awareness of radiation protection in patients and staff.

For years, CIRSE and its Radiation Protection Task Force have been campaigning to make interventional radiology a safe and well-accepted medical subspeciality. Since many interventional procedures involve use of ionizing radiation, CIRSE decided to focus not only on education on when and how to perform specific procedures, but also on radiation protection. Interventional radiologists are in the best position to promote radiation safety issues. But let´s face it, despite training in radiology many of us sometimes forget, ignore or misinterpret radiation safety issues. This special issue with a collection of articles on radiation protection tries to provide essential information on how to handle radiation in order to make patients and operators safe from unjustified and unnecessary high doses of radiation. This special issue is one of many attempts to convey the message that CT and angiographic equipment may be potentially dangerous for patients and operators if not used appropriately. Together with the authors of the articles from the special section, I believe that radiation safety issues should not be simply delegated to medical physicists and technologists. Radiation safety issues should be handled by a radiation safety team that includes interventional radiologists, who should also have a thorough understanding of radiation hazards and how to prevent them.

So, next time before you start a procedure, remember that there is always an invisible companion with you: ionizing radiation. If you know how to measure and deal with it, both you and your patients will be safe. This special issue aims to provide useful information for your daily practice, because together we can keep interventional radiology safe and make radiation-induced tissue reactions a part of the past.
